# Measurement of Cutting Temperature in Interrupted Machining Using Optical Spectrometry

**DOI:** 10.3390/s23218968

**Published:** 2023-11-04

**Authors:** Isaí Espinoza-Torres, Israel Martínez-Ramírez, Juan Manuel Sierra-Hernández, Daniel Jauregui-Vazquez, Miguel Ernesto Gutiérrez-Rivera, Felipe de Jesús Torres-Del Carmen, Tania Lozano-Hernández

**Affiliations:** 1 Departamento de Ingeniería Mecánica, División de Ingenierías, Campus Irapuato-Salamanca, Universidad de Guanajuato, Carretera Salamanca-Valle de Santiago km 3.5+1.8, Comunidad de Palo Blanco, Salamanca 36885, Mexico; i.espinozatorres@ugto.mx (I.E.-T.); miguel.gutierrez@ugto.mx (M.E.G.-R.); fdj.torres@ugto.mx (F.d.J.T.-D.C.); 2 Departamento de Ingeniería Electrónica, División de Ingenierías, Campus Irapuato-Salamanca, Universidad de Guanajuato, Carretera Salamanca-Valle de Santiago km 3.5+1.8, Comunidad de Palo Blanco, Salamanca 36885, Mexico; jm.sierrahernandez@ugto.mx (J.M.S.-H.); t.lozanohernandez@ugto.mx (T.L.-H.); 3 Centro de Investigación Científica y de Educación Superior de Ensenada (CICESE), División de Física Aplicada-Departamento de Óptica, Carretera Ensenada-Tijuana, No. 3918, Zona Playitas, Ensenada 22860, Mexico; jaureguid@ugto.mx

**Keywords:** cutting temperature, ratio pyrometer, milling, calibration system

## Abstract

This research presents an experimental study focused on measuring temperature at the tool flank during the up-milling process at high cutting speed. The proposed system deals with emissivity compensation through a two-photodetector system and during calibration. A ratio pyrometer composed of two photodetectors and a multimode fiber-optic coupler is employed to capture the radiation emitted by the cutting insert. The pyrometer is calibrated using an innovative calibration system that addresses theoretical discrepancies arising from various factors affecting the measurement of cutting temperature. This calibration system replicates the milling process to generate a calibration curve. Experimentally, AISI 4140 steel is machined with coated tungsten carbide inserts, using cutting speeds of 300 and 400 m/min, and feed rates of 0.08 and 0.16 mm/tooth. The results reveal a maximum recorded cutting temperature of 518 °C and a minimum of 304 °C. The cutting temperature tends to increase with higher cutting speeds and feed rates, with cutting speed being the more influential factor in this increase. Both the pyrometer calibration and experimental outcomes yield satisfactory results. Finally, the results showed that the process and the device prove to be a convenient, effective, and precise method of measuring cutting temperature in machine processes.

## 1. Introduction

The total rate of heat generation during machining is divided into three proportions: heat transported by the chip, heat conducted into the workpiece, and heat conducted into the tool [1,2,3]. The chip is considered waste produced during the cutting process; therefore, the central research focuses on measuring temperature, mainly in the tool, and secondly in the workpiece.

Machining with environmentally friendly systems is convenient, so it is necessary to improve the tools already in use. These tools were originally designed assuming they would be used in a conventional cooling environment. Currently, more eco-friendly systems are being developed, such as the minimum quantity lubrication (MQL) system. Additionally, tools with more resistant coatings capable of dry machining are being developed. Various studies have been conducted to analyze the appropriate conditions for dry machining or using an MQL system, such as the study by Abhishek Shukla et al. [4], who compared dry machining with an MQL system using soybean oil during a turning operation on AISI 304 steel. They concluded that using an MQL system resulted in optimized cutting forces and surface finish compared to dry machining. Rabinarayan Bag et al. [5] studied sustainable machining at high speeds using coated inserts in a dry environment on AISI 4340 steel. They used an ANOVA analysis to identify the cutting parameter that had the most influence on roughness, and found that feed rate was the parameter that most affected roughness. They also analyzed the factors influencing wear and concluded that cutting speed had the greatest influence on wear. A. Devillez et al. [6] studied cutting forces and wear in the dry machining of Inconel 718 with coated carbide tools. They tested different coatings (Uncoteded carbide K20, TiAlN, AlTiN, TiAlN+WC/C, TiAlN+MoST2+Ti) and concluded that the best coating was AlTiN, since wear was reduced due to its high hardness and ultra-fine crystallinity. Dry machining poses a challenge for tool designers, because it generates more friction and adhesion between the tool and the workpiece. This results in an increase in temperature, leading to tool wear. Therefore, several studies have been conducted to find the cutting temperature and develop more resistant tools. Zhang Shijun et al. [7] proposed a new approach to improve the prediction of the temperature of coated cutting tools, resulting in a model that can be used to design coated tools. Jingjie Zhang et al. [8] studied the effect of tool coating materials and coating thickness on the distribution of cutting temperature with coated tools, and observed that cutting temperature decreases with the increase in coating thickness of the same type. Moreover, they concluded that thermal conductivity and diffusion affect cutting temperature in transient and steady heat transfer. However, it is important to measure temperature in cutting processes, as it can establish better cutting conditions to increase tool life and reduce manufacturing costs. Furthermore, it can lead to improvements in cutting tools for more efficient machining and reductions in pollution generated in the processes.

Measuring cutting temperature during machining is essential because the temperature negatively affects tool performance and workpiece integrity. High temperature causes activation of tool wear mechanisms such as chemical and mechanical wear [9]. Tool wear causes bad surface finishing and a lack of precision in the final product. For instance, it was reported that the oxidation temperature of titanium nitride (TiN) coating on tools ranges from 450 °C to 700 °C [10]. The formed titanium oxide (TiO_2_) layer due to the interaction between titanium and oxygen in the tool–workpiece interface causes rapid wear on the tool [11]. The hardness of TiN undergoes a drastic reduction in its hardness over 400 °C. Another type of coating commonly used in cutting tools is titanium aluminum nitride (TiAlN). Although TiAlN coating has good stability at high temperatures, it reacts with hot air at 800 °C [12].

During machining operations, part of the heat is transferred to the workpiece. This may cause changes in surface hardness and even changes in the microstructure of the workpiece [13]. With the aim of increasing productivity, the tendency in machining operations is towards increasing cutting speed, feed rate and depth-of-cut [14]. Increasing these parameters causes an increase in cutting temperature. This situation brings progress in tool machining technology and cutting tools such as the development of new coatings. For these reasons, it is very important to measure cutting temperature during machining operations, and to maintain control of cutting conditions in order to reach a long tool life without sacrificing productivity.

Measuring temperature during the machining process is a challenging task for the following reasons:

- There is a relative movement between the workpiece and tool, and this motion usually occurs at high speed;
- The contact area and contact time between the tool and workpiece are small;
- Machining processes that involve chip formation occur in a severe environment which involves dirt, vibrations, and wetness (if cutting fluid is used);
- The shape of recently developed tools is complex;

Several methods that deal with temperature measurement during machining have been introduced, and others are still under development. In the literature, several methods of measuring this temperature can be found. For instance, Davies et al. [15] documented, from 234 papers, methods used for the measurement of temperature in material removal processes. According to Davies et al. [15], these methods can be ordered as follows: calorimetry, thermocouple, dynamic thermocoupling, spectral radiance thermometry, thermophysical, thermography, and ratio thermometry with an optic fiber and micro-resistance thermometer. One of the conclusions of this review was that milling processes have received less attention than turning, because they are more difficult. 

Leonidas et al. [16], in their comparative review paper of the different techniques available for the monitoring of cutting temperature, mentioned that in the case of milling applications, the response of the thermocoupling method may not be sufficient to measure sudden temperature change in high-speed milling operations. It is obvious that there is no general method that can be conveniently applied for all machining operations. However, in the case of interrupted machining at high cutting speed, it seems that radiation thermometry through optical fibers is suitable for measuring temperature at high speed. 

Gangwar et al. [17] reviewed optic fiber-based sensors for measuring temperature based mainly on different infrared sensors; most of them require that the fiber is at the measured temperature and is not used in machining applications. Li et al. [18] proposed a structure coated with composite materials. The sensors proposed measure in a range of 20–50 °C. This range limits application in the machining process because higher temperatures are expected. Abbasi et al. [19] used a photonic crystal fiber to measure the peak loss wavelength shift at different temperatures and establish a correlation between the two phenomena. The range of temperatures in this experiment is limited to 20–80 °C.

Díaz-Álvarez and Tapetado et al. [20,21,22] used a glass multimode optical fiber with a 62.5 µm diameter and a 0.275 numerical aperture (NA). The infrared (IR) energy was conducted through a wavelength division multiplexing (WDM) optic fiber filter that split the radiation into two spectral bands centered at 1.3 and 1.55 µm. Then, the IR radiation is collected using a dual indium gallium arsenide (InGaAs) photodetector. For calibration, a dry block calibrator black body kit (commercial equipment) was used. In a previous investigation, they found that temperature measuring is insensitive to the fiber position if the target surface is larger than the spot projected by the NA fiber on the measuring surface. 

Sutter et al. [23] used an intensified charge-coupled device (CCD) camera with a glass arrangement to measure the cutting temperature during orthogonal machining. The calibration was carried out with a blackbody. According to the calibration curve, the device measures temperature over 650 °C.

Ueda et al. [24] developed an IR pyrometer to measure temperature during the grinding of carbon steel. An optical condenser was utilized to focus the irradiance to a single indium arsenide (InAs) photodetector. The signal was amplified and filtered, then recorded in a synchroscope. The calibration system was discussed shortly. In general, the fiber was set in front of the workpiece at about 0.5 mm; at the same time, the temperature of a specimen was checked using a C-A thermocouple. In 2008, Ueda et al. [25] developed a new type of pyrometer in which the optical fiber was set through a rotating tool (for the milling process) or a rotating workpiece (for the turning process). A non-contact fiber coupler was used to transmit the infrared rays to a two-color pyrometer composed of an indium antimonide (InSb) cell and a InAs cell. 

Davies et al. [26,27] reported the development of an IR microscope. The system was based on a commercially available InSb focal plane array sensitive to radiation in the 3 µm and 5 µm wavelength range. The system was calibrated against a miniature blackbody. The temperature of the blackbody was measured using a type-S thermocouple. The temperature was varied in intervals of 100 °C to 700 °C. Since a single photodetector was used, an analysis of the behavior of emissivity as a function of temperature was presented. 

Jehnming Lin et al. [28] used a single photodetector lead sulfide (PbS) sensor to measure temperature in the turning of AISI 1045. The infrared rays were conducted through a zirconium optical fiber of 240 µm in diameter with a numerical aperture of 0.2 The cut-off wavelength was about 3µm. The setup of this experiment was intended to measure the rake face; however, due to the location of the optical fiber (5 mm), it is more likely that the temperature on the free side of the chip was measured instead of the work-chip contact area. In this investigation, there was not a detailed description of the calibration system.

Han et al. [29] presented a two-color pyrometer which was used in the turning of AISI 316L. The arrangement consisted of a multimode optical fiber which was inserted into the tool insert until it reached 1 mm from the bottom face inside the micro hole. The IR rays are directed to the plano-convex lens to convert them into parallel rays, then the rays are split into two identical rays by a beam splitter. Two band filters were used(Thorlabs FB2000-500 and FB2500-500) which means that the pass center wavelength is 2 µm and 2.5 µm, respectively, both with a bandwidth 0.5 µm. Two InGaAs-amplified photodetectors were used to transform the radiant energy into an electrical signal. The cutting temperature was not directly measured on the rake face, but some distance beneath the tool. The calibration procedure was carried out with a commercial IR calibrator (fluke 9173) with a stable emissivity. It was argued that if the wavelength measurement is narrow and close, the assumption of a constant emissivity is reasonable, and not dependent on the wavelength. 

Al Huda et al. [30] used a two-color pyrometer to measure the temperature of tool–chip interface in turning AISI 1045. In this investigation, the claim of measuring temperature in the interface of the tool–chip (rake face) was strong due to the use of translucent alumina. In this way, the zone of contact was in the view of the optic fiber. Two photodetectors with different spectral responses were used in this investigation. The semiconductors used as photodetectors were germanium (Ge) and indium antimonide (InSb). The optic fiber used was quartz (SiO_2_). A Ge filter was added to the InSb cell to reduce the short wavelength of the IR rays. The calibration system consisted of a heating element that heats the workpiece, while the optic fiber was set inside of the alumina tool. During the heating, the temperature was recorded with a Pt/Pt-Rd thermocouple. The authors argued that if the measurable wavelength of both detectors is close enough, it is reasonable to assume that the emissivity of the object is constant and not dependent on the wavelength. 

Yashiro et al. [31] measured the cutting temperature when end-milling CFRPs. They used the thermocoupling method and a commercial IR camera. Dynamic thermocoupling was used to measure temperature in the cutting point, an embedded thermocouple was used to measure the transition temperature in the polymeric matrix, and the surface temperature of the tool was measured with a commercial IR thermograph camera. 

There is an intrinsic steep gradient during machining processes in very small areas. For this reason, it is very difficult to see or distinguish in a commercial device the actual maximum temperature in the cutting zone. However, due to the importance of cutting temperature in research into tool technology, some researchers [32,33,34] have used commercial IR cameras in order to evaluate cutting temperature. Hijazi et al. [35] used a commercial camera to measure temperature in the orthogonal cutting of aluminum 6061-T6. In this investigation, a procedure was developed to compensate for the emissivity. Valiorgue et al. [36] used an FLIR camera to measure temperature in orthogonal cutting. In this research work, the emissivity was compensated using the ratio between the blackbody theoretical luminance and the measurements obtained directly from the sample. Due to the experimental set up, the temperature could exceed 550 °C. Kus et al. [37] used a commercial infrared pyrometer with a K-type thermocouple to measure temperature during the turning of AISI 4140 with coated carbide tools. The variation in emissivity reported in the paper was wide (0.45–0.85). They claimed that the tool–chip interface was measured, but due to the measuring conditions, the measurements were taken 45 cm away from the point of view of the chip; thus, the interface between the chip and rake face was hidden. Davoodi and Hosseinzadeh [38] claimed that their system can measure the heat transferred to the workpiece during the high-speed machining of bronze alloys. In this investigation, face-milling was used as a machining process. In the research work of Davoodi and Hossenzadeh, there were no details about the type of sensors used nor the calibration procedure. 

The research published up to now is focused mainly on continuous cutting, such as turning operations. Furthermore, the calibration procedure was frequently carried out with commercial equipment based on the supposition of a black body. Therefore, there is no compensation for the emissivity during calibration. The common assumption used in previous research work is that if the spectral range of the photodetectors is close enough, and there is a less sizeable departure from the black body, and therefore the error due to emissivity is not considerable. 

In this work, a new configuration of a near-infrared (NIR) pyrometer system is presented. This system is intended to be used in high-speed milling operations wherein a fast response and reliability is needed. The proposed calibration system imitates the conditions of interrupted signal from the tool to the photodetectors, such as happens in milling operations; therefore, the calibration is carried out in similar conditions. At the same time, the proposed system compensates for emissivity, since the actual cutting tool is used during the calibration. Therefore, the error due to the assumption of a gray body is reduced. In the experiments, AISI 4140 was used as a workpiece material, and the tool was a TiAlN-coated carbide. The optical fiber was set in such a way that the temperature in the flank face is measured. The flank face is the zone of the tool that is in contact with the workpiece; the wear in this spot is commonly used as a criterion for determining tool life. The results show good repeatability in both calibration, procedure, and measuring of cutting temperature. 

## 2. Materials and Methods

### 2.1. Background Theory

The emission of electromagnetic radiation by an object is the consequence of its ab- solute temperature, and covers a range from the visible to the infrared spectrum. The emission is directly associated with the temperature of the object. Planck’s law describes the spectral distribution of the intensity of electromagnetic radiation emitted by a black body, and is expressed by the following equation [39]:

(1) $$E_{BB}\left(\lambda ,T\right)=\frac{2hc^{2}}{\lambda^{5}}{\left(e^{\frac{hc}{\lambda kT}}-1\right)}^{-1}$$

where *h* represents the Planck constant, *c* is the speed of light in a vacuum, *λ* is the wavelength, *k* is the Boltzmann constant, and *T* is the absolute temperature in Kelvin. In the case of real bodies, radiance is composed of the sum of the absorption, transmission, and reflection processes. The relationship between the radiance of a body at a given temperature and the radiance of a black body at the same temperature is known as emissivity, and is expressed by the following equation [40]:

(2) $$\;\varepsilon =\frac{E_{\lambda }(\lambda ,T)}{E_{BB,\lambda }(\lambda ,T)}$$

However, in most practical scenarios, a material’s emissivity is influenced by various factors, such as temperature, wavelength, observation angle, and surface conditions. One approach to obtaining temperature estimations without the need to know the material’s emissivity is through measuring the ratio *R* between the radiances ($E_{\lambda_{1}}$ and $E_{\lambda_{2}}$) emitted by the same object at two different wavelengths [41].

(3) $$\;R=\frac{E_{\lambda_{1}}(\lambda ,T)}{E_{\lambda_{2}}(\lambda ,T)}$$

The wavelengths used for the measurement are close to each other; thus, we assume that the emissivity at both wavelengths is equivalent ($\varepsilon_{\lambda_{1}}$=$\;\varepsilon_{\lambda_{2}}$). Therefore, the ratio can be calculated as follows [42].

(4) $$\;R=\frac{\lambda_{2}^{5}\varepsilon_{\lambda_{1}}}{\lambda_{1}^{5}\varepsilon_{\lambda_{2}}}\left[\frac{e^{\frac{hc}{\lambda_{2}kT}-1}}{e^{\frac{hc}{\lambda_{1}kT}-1}}\right]$$

Consequently, it is possible to determine the temperature using the values of the radiance ratio:

(5) $$\;T=\frac{hc}{k}\left[\frac{\frac{1}{\lambda_{2}}-\frac{1}{\lambda_{1}}}{\ln\left(R\right)-\ln\frac{{\lambda_{2}}^{5}}{{\lambda_{1}}^{5}}}\right]$$

### 2.2. System

**Figure 1:**
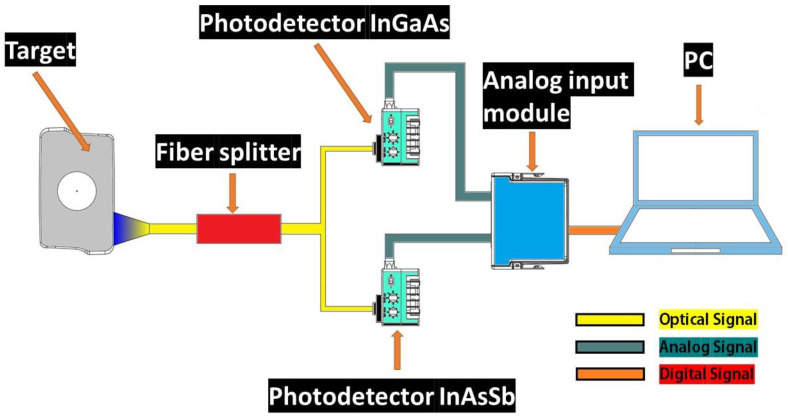
Schematic diagram of the fundamental structure of the ratio pyrometer.

The pyrometer’s diagram is presented in Figure 1. The IR radiation emitted by the target, arising from its temperature, is collected by the optical fiber and transmitted through the optical fiber splitter. The optical fiber splitter (Thorlabs Inc., Newton, NJ, USA TM200R2S2B) divides the signal into two signals with varying power levels: 10% of the energy is directed to an InGaAs photodetector (Thorlabs Inc., Newton, NJ, USA PDA10DT), while the remaining 90% is directed to an InAsSb photodetector (Thorlabs Inc., Newton, NJ, USA PDA10PT). The optical signals are converted by the photodetectors into electrical signals, which are then received by the Data Acquisition (DAQ) card at a sampling frequency of 20,000 S/s.

A multimode optical fiber was employed due to its ability to guide light with multiple transverse guided modes for a given optical frequency. Photodetectors with low noise-equivalent power (NEP = 2.11 pW/$\sqrt{Hz}$) were implemented. A low NEP value is desirable as it corresponds to reduced background noise, leading to a more sensitive detector and enabling the measurement of temperatures in a range commonly found in machining operations. The signal processing was focused on deriving the ratio between the two signals captured by the photodetectors (InGaAs/InAsSb), thereby determining the temperature. The characteristics of the optical fiber and photodetectors are presented in Table 1.

### 2.3. Frequency Characteristics of Photodetectors

Accurately measuring temperature during milling processes is quite challenging due to the high and variable tool rotation speed, which is dependent on the cutting parameters. Given that milling involves intermittent cutting, the inserts on the tool holder enter and exit the work material with each revolution. Consequently, the signal captured by the optical fiber appears as a pulsed signal throughout the entire cutting process, and its frequency varies according to the cutting speeds employed.

**Figure 2:**
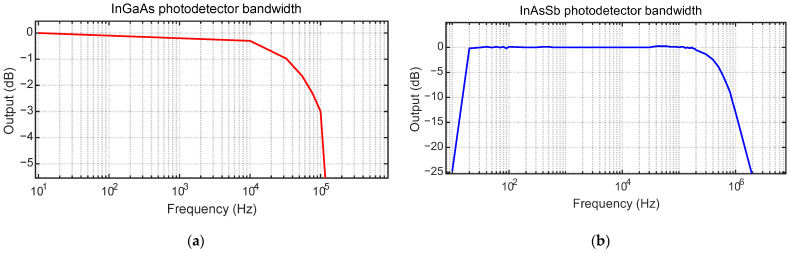
Bandwidth of the photodetectors (data supplied by Thorlabs): (**a**) InGaAs photodetector (**b**) InAsSb photodetector.

The bandwidth and gain used for the InGaAs photodetector are 50 kHz and 40 dB, respectively, and for the InAsSb photodetector are 16 kHz and 60 dB, respectively. Low bandwidths were chosen for the photodetector capabilities, because there is less noise at the output of the photodetectors and the system captures low frequencies of around 100 Hz. On the other hand, the amplification gain used is higher for the InAsSb photodetector, since it was observed that it showed a lower sensitivity to the radiance obtained from the insert. The frequency response of the photodetectors is depicted in Figure 2. It is evident that the InGaAs photodetector (Figure 2a) demonstrates a flat response for sinusoidal signals in the range of 0 to 10 kHz. On the other hand, the InAsSb photodetector (Figure 2b) exhibits a flat response within a frequency range of 30 to 100 kHz. Given that the frequencies utilized during the experiments fall within these flat response ranges, the captured signal will not experience significant losses and will be independent of the tool rotation speed. Consequently, any fluctuations in the cutting speed will not impact the accuracy of the measurements performed by the pyrometer.

### 2.4. Experimental Procedure and Conditions

**Figure 3:**
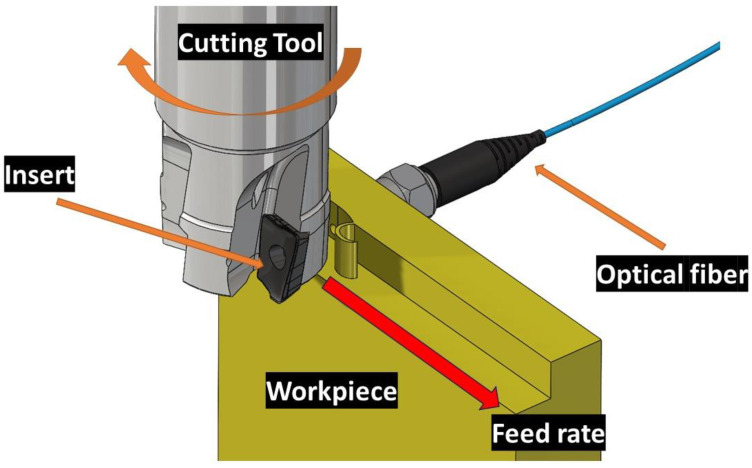
Schematic picture of the shoulder-milling process and optical fiber location during the experiments (the axial cutting depth used is 4 mm, and the radial depth is 1 mm).

The experimental set-up is shown in Figure 3. In this study, a horizontal CNC milling machine (VIWA, Guadalajara, Mexico, model VF4B M400) was employed to conduct a shoulder-milling process using an up-milling strategy.

**Figure 4:**
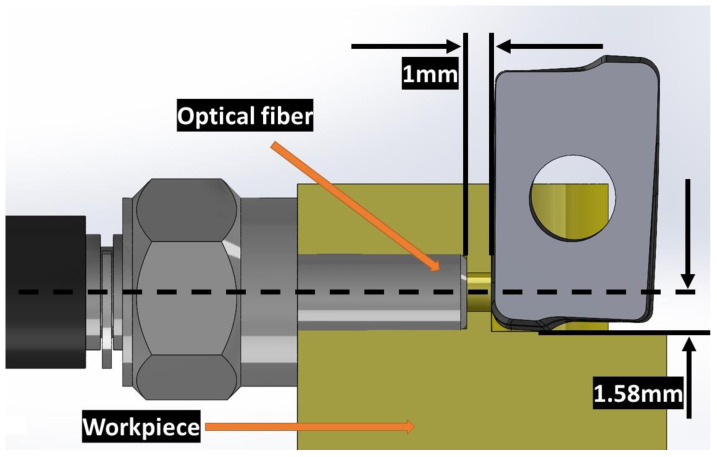
Set up of the optic fiber in the workpiece and its position with respect to the tool. The optic fiber is set in front of the flank face of the tool.

In Figure 3, the workpiece is fixed on the table of the machining center while the cutting tool turns. The relative movement of the workpiece is opposite with respect to the rotation of the cutting tool. In the opposite side of the cutting, the optical fiber is set inside a fine hole. During this process, cutting temperatures were measured and analyzed. For the milling operations, tungsten carbide inserts coated with TiAlN (Iscar, Migdal Tefen, Israel, HM90 APKT 1003PDR) were used, which were mounted onto a specific insert holder ((Iscar, Migdal Tefen, Israel, HM90 E90A-D25-4-C25). In each experimental run, a single insert was mounted on the insert holder. The workpiece material chosen was AISI 4140, with dimensions of 10 mm thickness, 50 mm height, and 64 mm width. As shown in Figure 4, the optical fiber is precisely set in such a way that the distance is always keep at 1 mm away from the cutting tool and is directed to the flank face of the insert. The workpiece material featured a small hole (approximately 1.58 mm in diameter) extending towards the machining zone. This hole was included to facilitate the capture of electromagnetic waves emitted by the insert through an optical fiber. 

The optical fiber, approximately 1 m in length, was affixed to the system via a hole measuring 3.17 mm in diameter. This arrangement ensured the stability of the optical fiber, and at the same time minimized any potential motion that could interfere with measurements. The distance between the rotating insert (responsible for material removal) and the stationary optical fiber was maintained at a consistent 1 mm, as shown in Figure 4. Temperature measurements were conducted on the cutting flank in the designated region. The cutting conditions employed in the experiment are outlined in Table 2.

#### Wear

In machining operations, cutting tools are confronted with conditions that challenge their integrity, such as cutting forces and friction, generating high temperatures due to direct contact between the tool and the workpiece. Consequently, workpieces may undergo alterations and experience negative effects on their surface finish. Moreover, tools are subjected to changes in their mechanical properties, which can trigger various wear mechanisms. Since the central objective of this research revolved around the precise measurement of temperature during the machining process, it was imperative to verify that wear did not influence the temperature obtained.

**Figure 5:**
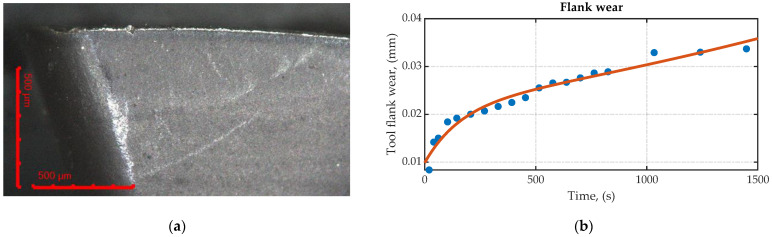
Tool wear monitoring: (**a**) flank wear at 1500 s of cutting time; (**b**) Wear curve. Cutting speed: 300 m/min, feed rate: 0.16 mm/tooth; and cutting depth: 4 mm.

The cutting conditions used during the tool wear test were as follows: a cutting speed of 300 m/min, a feed rate of 0.16 mm/tooth, and a cutting depth of 4 mm. The wear tests demonstrated that the cutting tool exhibited adequate resistance to the cutting conditions used in the experimentation, complying with the flank wear limit established by the ISO standard [43], which is 0.3 mm. Figure 5b presents a graph showing a wear of 0.035 mm after 1500 s of machining using the aforementioned parameters. The wear test did not extend to the failure region, as the primary objective was to demonstrate that wear does not affect temperature measurement in the experiments conducted in this article. The graph displays a typical wear trend, with a break-in period and a steady-state wear region. In Figure 5a, an insert is shown, examined under a microscope at 10× magnification and revealing a flank wear of 0.035 mm. It is also observed that some areas exhibit chipping wear. Tests were conducted for a total time of 200 s and a flank wear of 0.02 mm, approximately; therefore, the wear experienced in this study can be considered negligible in terms of its contribution to the temperature increase.

### 2.5. Calibration

**Figure 6:**
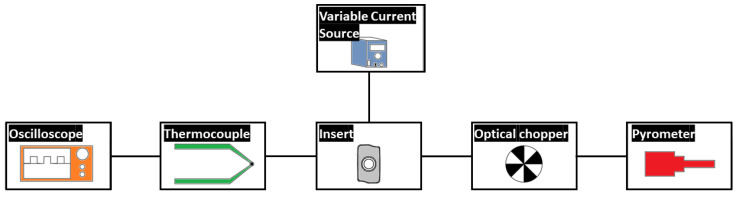
Diagram of the calibration system.

A new calibration system is introduced. The actual tool used in the milling process is heated to high temperatures, and the electromagnetic radiation emitted by this material is captured by an optical fiber. The diagram of the calibration system can be seen in Figure 6.

To carry out this calibration, a previously calibrated K thermocouple is utilized. This system consists of a type-K thermocouple sensor connected to a high-precision Yokogawa module; its main characteristics are shown in Table 1. The thermocouple is capable of measuring temperatures up to 982 °C. The oscilloscope is equipped with a calibrated module that enables data acquisition at a sampling rate of 500 S/s.

**Figure 7:**
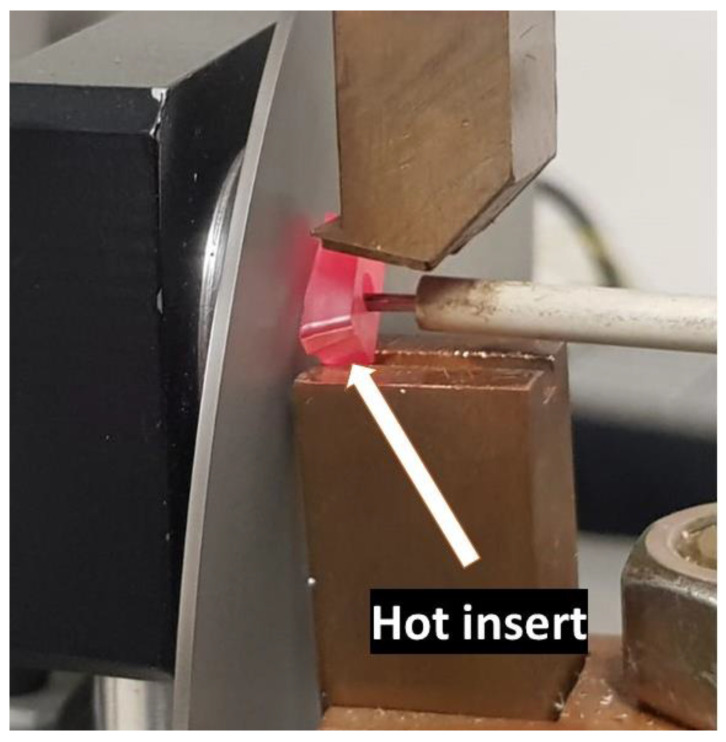
Insert heated to 506 °C.

For heating the tool, the Joule effect is employed. The heated tool is shown in Figure 7. A variable direct current source is used, ranging from 20 to 200 A. The signal emitted by the tool undergoes discretization through an optical chopper, aiming to simulate the intermittent cutting operation characteristic of milling. The obtained signal is processed to derive a calibration curve.

**Figure 8:**
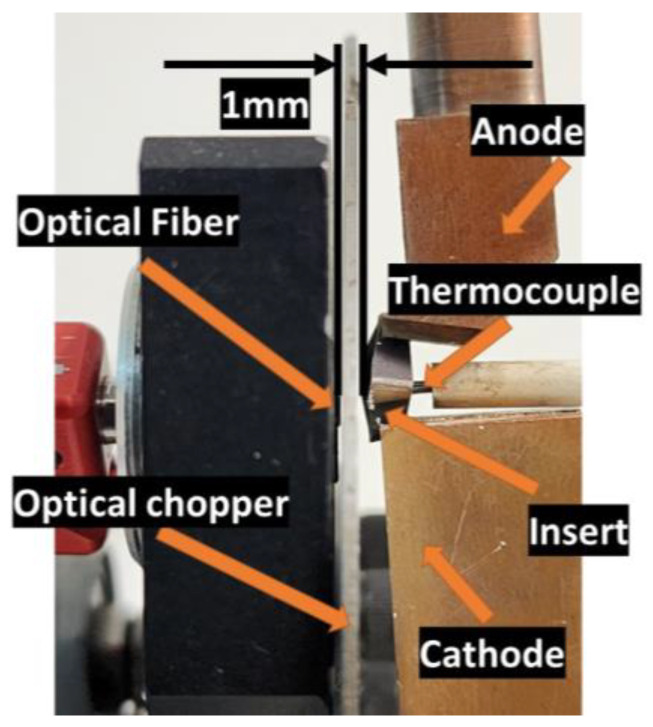
Illustration of the adjustment of the optical fiber in the calibration system.

During calibration, effort was made to faithfully replicate the machining process. The frequency ranges used in machining were similarly adjusted in the calibration system. The incidence face of the optical fiber and the insert were aligned at an approximate distance of 1 mm, as shown in Figure 8. 

Ueda et al. considered a scenario wherein the object had a radius of 0.1 mm and was held at a constant temperature of T = 2000 °C. Two types of pyrometers were employed: one based on two-color measurement, and the other on InSb-IRP. The manipulated variable was the measurement distance between the object and the optical fiber receiving face. It was observed that at critical distances less than 1 mm, the InSb-IRP pyrometer yielded inaccurate measurements, while the ratio pyrometer maintained a temperature accuracy of 2000 °C up to distances of 10 mm. A two-color pyrometer can determine temperature independently of the measurement distance or object size [44]. 

**Figure 9:**
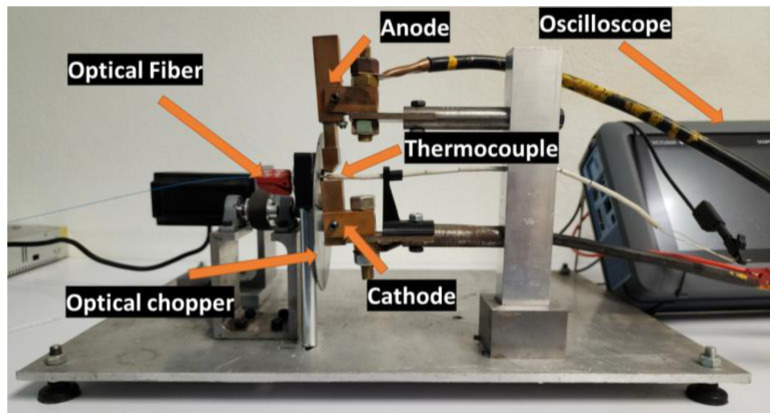
Calibration system.

The thermocouple was aligned with the central axis of the optical fiber to carry out temperature measurements under the same projection as the thermocouple’s contact area. The calibration system is shown in Figure 9. The electrodes of this system were manufactured from copper to prevent heating and power loss. The anode is equipped with a compression spring that dissipates forces due to thermal expansion, thereby ensuring that the insert remains in a constant position. This stable design of contact between the insert and the electrode prevents the generation of sparks caused by insufficient contact. The optical chopper features a rotating axis that is coupled to a pair of bearings, ensuring the stability of the axis and preventing any contact between the rotating axis and the system’s electrodes. The rotating axis contains four slots, each with a thickness of 1.58 mm.

## 3. Results

### 3.1. Calibration Curve

During the calibration process, a controlled electric current was applied to the insert for a duration of 1 min. The aim was to achieve uniform heating of the insert, effectively reducing any significant temperature gradient. This approach ensured consistent temperature distribution across all points within the insert. Subsequently, a series of eight incremental tests were conducted, commencing from 268 °C and progressing until reaching 652 °C. The output from the InGaAs photodetector for each test was analyzed to statistically verify its variability in order to demonstrate that the temperature in the insert remained constant. The InGaAs photodetector was exemplified because the InAsSb photodetector showed similar results. The statistical analysis is presented in Table 3. A minimum standard deviation of 0.38 °C was recorded at a temperature of 415 °C, while a maximum of 1.37 °C was observed at a temperature of 652 °C. However, the data dispersion suggests a significant temperature uniformity across all the tests.

**Figure 10:**
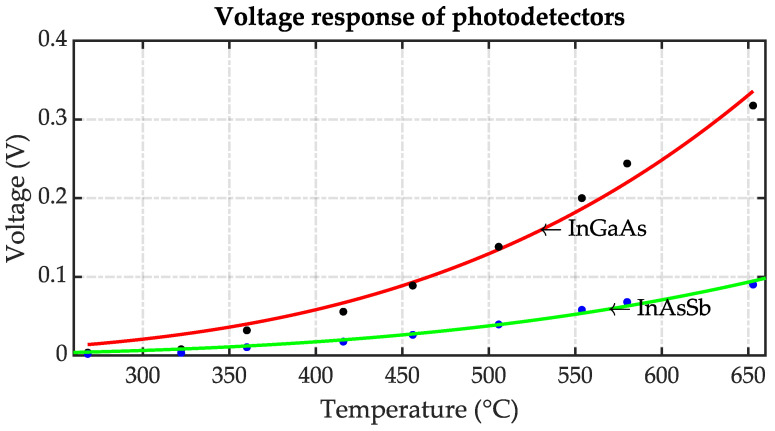
Voltage response of photodetectors.

An increase in the output voltage is observed as the temperature rises, as illustrated in Figure 10. Although each photodetector exhibits a similar trend, the InGaAs photodetector stands out with a more pronounced voltage response compared to its InAsSb counterpart. To fit the data obtained in the calibration tests, a polynomial model was applied, $f\left(x\right)=ax^{b}$. The result was a highly reliable fit, supported by R-squared values of 0.9823 for the InGaAs photodetector data and 0.9868 for the InAsSb photodetector voltages. 

**Figure 11:**
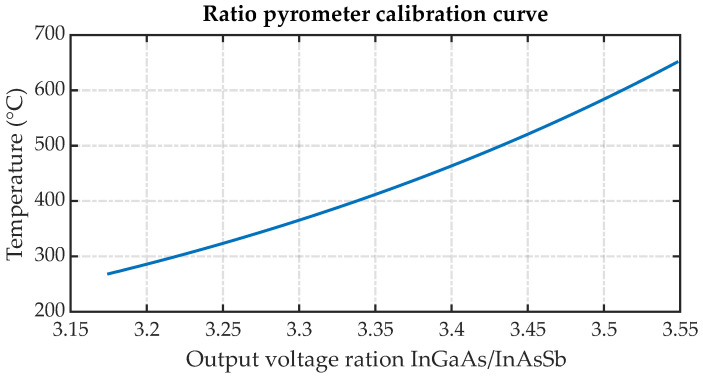
Ratio pyrometer calibration curve.

The pyrometer designed in this study demonstrates the ability to record temperatures ranging from a minimum of 268 °C to a maximum of 652 °C. The minimum temperature that the photodetector can measure is primarily limited by its sensitivity and the noise-equivalent power (NEP). However, the maximum temperature is constrained only by the calibration source of direct current used to heat the insert, as photodetectors have a wide range for saturation. The calibration curve presented in Figure 11 emerges as a result of differences in the spectral response of the photodetectors. This calibration curve was obtained using an insert with the same characteristics as used in the experimentation.

**Figure 12:**
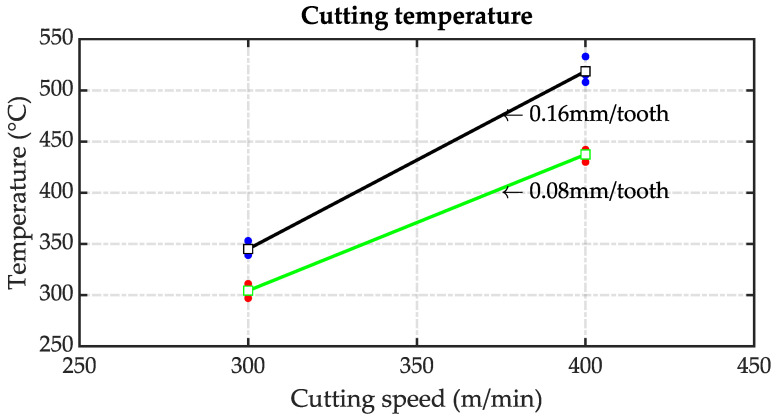
Influence of cutting speed and feed on temperature.

The influence of cutting conditions on the temperature at the flank face is investigated. The impact of an increase in cutting speed and feed rate is shown in Figure 12. In the plot shown in Figure 12, the dots represent the repetitions in each condition and the square represents the mean value. It is evident that both cutting parameters have a significant influence on the temperature increase. The temperature is 304 °C at a cutting speed of 300 m/min, and increases to 437 °C at 400 m/min with a constant feed rate of 0.08 m/tooth. If the feed rate is increased to 0.16 m/min, a temperature of 345 °C is recorded at a speed of 300 m/min, and it increases to 518 °C at 400 m/min. The dots represent the replica, and the squares the mean value in each condition. 

### 3.2. Design of Experiments

The essence of experimental design lies in identifying the factors that exert the most significant influence on the target output variable under analysis. Machining temperature, for instance, can be influenced by a multitude of factors. In this experiment, the factors under consideration encompass cutting speed and feed rate. An experimental factorial design called 2^2^ was adopted, comprising twelve experiments involving two distinct factors, two different levels of experimentation, and three repetitions for each experiment. This type of factorial design is known as an optimal design because it minimizes the variance of the model regression that is used to construct the response surface. This forms part of the response surface methodology. Since this method is based on statistics, it is useful to draw objective conclusions about the influence of the factors (cutting speed and feed rate) on the response (temperature). The factors and corresponding levels are outlined in Table 4. 

A study was conducted to ascertain which of these effects hold statistical significance. This implies the assertion, with a low probability of error, that a factor truly influences a given response.

An analysis of variance (ANOVA) was chosen for this purpose. This statistical method is precise and formal, involving the allocation of total variance to factors, thus enabling statistical tests to determine, with a specific level of confidence, the factors that significantly influence the response. The results of the variance analysis are presented in Table 5, revealing that both cutting speed and feed rate significantly influence the temperature increase. This conclusion is supported by their p-values being less than 0.05, indicating statistical significance. The mean square of the error represents an estimate of the variance. Therefore, it is estimated that the standard deviation of the experiments is equal to 8.7 °C.

**Figure 13:**
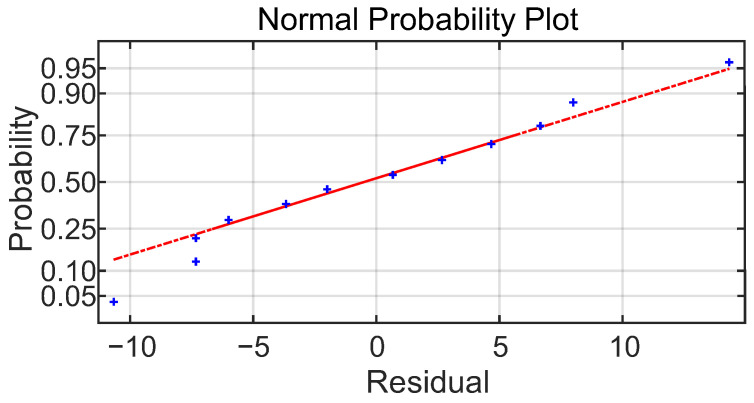
Normal probability plot of residuals.

The model adequacy is carried out from the analysis of the residuals. The residuals are calculated from the difference between the predicted values from the fit model and the actual experimental data. The normal probability plot of residuals is shown in Figure 13.

The behavior of residuals looks like a sample of normal distribution, close to a linear behavior in a normal scale; therefore, the assumption about the normal distribution of the error is fulfilled. 

The *R_adj_*^2^ can be used as an indicator of the proportion of the variability in the data explained by the model. *R_adj_*^2^ is defined as:

(6) $$\;{R_{adj}}^{2}=1-\frac{{SS}_{E}/{DOF}_{E}}{{SS}_{Total}/{DOF}_{Total}}$$

where *SS_E_* is the sum of squares of the error, *DOF_E_* is the degrees of freedom of the error, *SS_Total_* is the total sum of squares, and *DOF_Total_* is the total degrees of freedom. Applying the numerical values shown in Table 5 results in *R_adj_* = 0.989. This means that the proportion of total variability is well explained by the model [45]. 

#### 3.2.1. Equation of Effects

An empirical prediction model is constructed. This equation elucidates the relationship between the response and the pertinent factors (and interactions). The model facilitates the estimation of a response under non-experimental conditions, although limited to the experimental range. The regression equation derived from the significant effects is illustrated in Equation (6).

(7) y=401.33+76.667A+30.5B+10.167AB

#### 3.2.2. Surface and Contour Plots 

**Figure 14:**
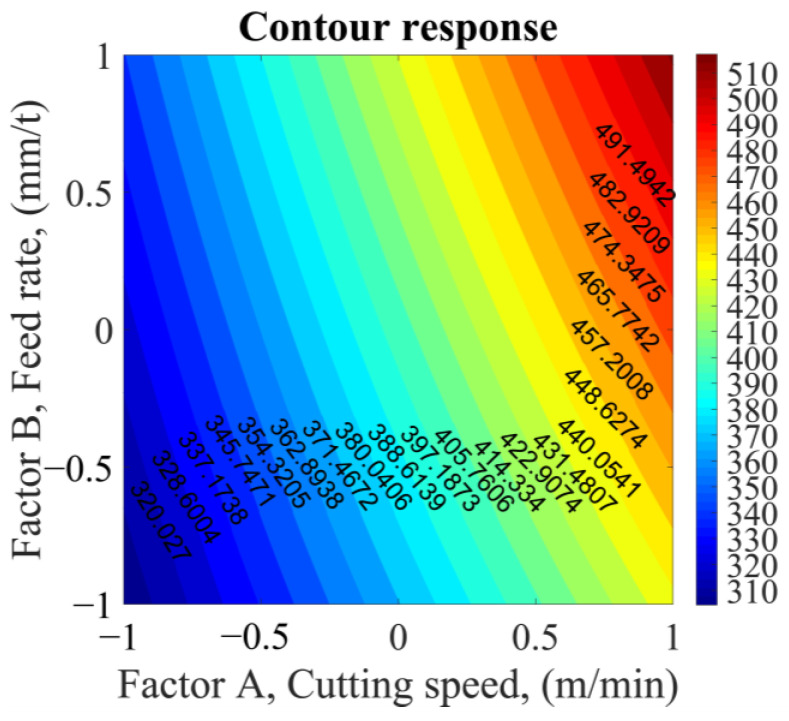
Contour response.

Contour plots and surface plots are displayed in Figure 14, illustrating the relationship between temperature (output variable), feed rate, and cutting speed (two predictor variables). The highest temperature is achieved with higher feed rate and cutting speed values, while the lowest temperature is observed at lower levels, as expected. 

## 4. Conclusions

This article introduced a ratio pyrometer for measuring cutting temperatures at the tool interface. Experiments were conducted in face-milling to validate the pyrometer’s functionality. To calibrate the pyrometer, a novel calibration system was developed. The cutting tool was used as the target, and the milling process was replicated. The main conclusions are as follows:

1. The ratio pyrometer can measure cutting temperatures at specific points on the tool due to its small sensing area. The measured temperatures are independent of the material’s emissivity. The minimum measurable temperature is 268 °C, and the maximum is limited only to the heating device used in the calibration procedure.
2. The trend in the results reveals that higher cutting temperatures are associated with elevated cutting speeds and feed rates at the cutting edge during milling. The cutting temperature increased linearly from 304 °C to 518 °C. However, the cutting speed exerts a greater influence on the temperature. The estimated standard deviation during the machining experiments was 8.7 °C.
3. A calibration system was constructed and tested to heat cutting inserts, with the system achieving maximum temperatures of 650 °C with a standard deviation of only 1.37 °C. The high fidelity with which the calibration system replicated the cutting process facilitated the acquisition of a precise and realistic pyrometer calibration.

## Figures and Tables

**Table 1:** Characteristics of measuring instruments.

Optical fiber	Core material: Pure Silica
	Cladding Material: Fluorine-Doped silica
	Core Diameter: 200 µm Cladding Diameter: 220 µm NA: 0.22 Wavelength Range: 0.4–2.4 µm
InGaAs photoconductive detector	Wavelength Range: 0.9–2.57 µm Noise-Equivalent Power (NEP): 2.11 pW/Hz^1/2^ @ DC—2.5 kHz (for 70 dB Gain and 1 MHz Bandwidth) Gain Setting: 0, 10, 20, 30, 40, 50, 60, 70 dB Bandwidth Setting: 500 Hz–1 MHz. Peak Wavelength: 2.3 µm Peak Responsivity: 0.8–1.6 A/W
InAsSb photoconductive detector	Wavelength Range: 1.0–5.8 µm Noise-Equivalent Power (NEP): 1.49 x10^−10^ W/Hz^1/2^ (for 40 dB Gain and 1600 kHz Bandwidth) Gain Setting: 0, 4, 10, 16, 22, 28, 34, 40 dB Bandwidth Setting: 12.5–1600 kHz. Peak Wavelength: 4.9 µm Peak Responsivity: 1.3 A/W
Thermocouple	Type: K
	Maximum Temperature: 982 °C
	Diameter: 0.81 mm

**Table 2:** Cutting conditions.

Machine tool	Shoulder milling
	Diameter = 25 mm
	Insert: HM90 APKT 1003PDR IC98 PVC coated carbide (TiAlN)
Machine	CNC vertical machining center, VIWA Guadalajara, Mexico/model VCM 1050 M400ACT
Workpiece	Carbon steel (AISI 4140)
Cutting parameters	Spindle revolution: 5092–3819 rpm
	Cutting speed: 300–400 m/min
	Feed rate: 305.57–814.72 mm/min
	Feed per tooth: 0.08–0.16 mm/tooth Radial depth of cut: 1 mm Axial depth of cut: 4 mm
Environment	Dry

**Table 3:** Descriptive statistics of each test for the InGaAs photodetector.

Test According to the Measured Temperature	268 °C	321 °C	360 °C	415 °C	457 °C	506 °C	553 °C	600 °C	652 °C
Standard deviation (°C)	1.46	1.36	0.48	0.38	0.94	1.02	0.58	0.93	1.37
Variance (${{}^{\circ}C}^{2}$)	2.14	1.86	0.23	0.14	0.88	1.05	0.33	0.86	1.90

**Table 4:** Parameters and their levels.

Factor	Levels	
	1	2
A, Cutting speed V, (m/min)	300	400
B, Feed rate f, (mm/tooth)	0.08	0.16

**Table 5:** ANOVA for temperature.

Source of Variation	Degree of Freedom	Sum of Squares	Mean Square	$F_{0}$	*p*-Value
A (cutting speed)	1.0	70,533.33	70,533.33	913.05	1.56 × 10^−9^
B (feed rate)	1.0	11,163.00	11,163.00	144.50	2.12 × 10^−6^
AB	1.0	1240.33	1240.33	16.06	3.9 × 10^−3^
ERROR	8.0	618.00	77.25	1.00	
TOTAL	11.0	8554.67	7595.88	98.33	

## Data Availability

Under suitable request.
